# Rice phenolamindes reduce the survival of female adults of the white-backed planthopper *Sogatella furcifera*

**DOI:** 10.1038/s41598-020-62752-y

**Published:** 2020-04-01

**Authors:** Wanwan Wang, Zhuoxian Yu, Jinpeng Meng, Pengyong Zhou, Ting Luo, Jin Zhang, Jun Wu, Yonggen Lou

**Affiliations:** 1 Department of Chemistry, Zhejiang University, Hangzhou, 310058 China; 2 State Key Laboratory of Rice Biology & Ministry of Agriculture Key Lab of Molecular Biology of Crop Pathogens and Insects, Institute of Insect Sciences, Zhejiang University, Hangzhou, 310058 China

**Keywords:** Plant stress responses, Herbivory

## Abstract

In response to infestation by herbivores, rice plants rapidly biosynthesize defense compounds by activating a series of defense-related pathways. However, which defensive compounds in rice are effective against herbivores remains largely unknown. We found that the infestation of white-backed planthopper (WBPH) *Sogatella furcifera* gravid females significantly increased levels of jasmonic acid (JA), jasmonoyl-isoleucine (JA-Ile) and H_2_O_2_, and reduced the level of ethylene in rice; levels of 11 of the tested 12 phenolamides (PAs) were subsequently enhanced. In contrast, WBPH nymph infestation had no effect on levels of JA, JA-Ile, ethylene and H_2_O_2_ in rice, and enhanced levels of only 2 of 12 PAs. Moreover, infestation by brown planthopper *Nilaparvata lugens* gravid females also affected the production of these PAs differently. Bioassays revealed that 4 PAs – N-feruloylputrescine, N-feruloyltyramine, feruloylagmatine and N1,N10-diferuloylspermidine – were toxic to newly emerged WBPH female adults. Our results suggest that WBPH- or BPH-induced biosynthesis of PAs in rice seems to be shaped primarily by the specific profile of defense-related signals elicited by the herbivore and that PAs play a role in conferring the resistance to WBPH on rice.

## Introduction

When attacked by herbivores, plants recognize herbivore-associated molecular patterns and danger-associated molecular patterns and activate multiple signaling pathways mainly mediated by mitogen-activated protein kinase (MAPK) cascades, jasmonic acid (JA), jasmonoyl-isoleucine (JA-Ile), salicylic acid (SA) and ethylene^1–3^.The activated signaling pathways lead to the production of defensive compounds, which in turn enhance the resistance of plants to herbivores^4^.

Phenolamides (PAs), also known as phenylamides or hydroxycinnamic acids amides, are a large class of plant secondary metabolites. They are conjugates of various hydroxycinnamic acids (coumaric, caffeic, ferulic acids, among others) with mono/polyamines (spermine, putrescine, tyramine, among others). In addition to playing roles in plant growth and development (i.e., flower development, senescence, sexual differentiation and cell division^5–7^), PAs have been proven to play important roles in plant defenses^8,9^. PAs, for example, accumulate in the cell wall following pathogen infection, and are known to have antifungal and antibacterial activities^10,11^. Recently, it was observed that potato, when inoculated with *Phytophthora infestans*, increased the production of several kinds of PAs, including N-feruloylputrescine (FerPut), p-coumaroyltyramine (CouTyr), N-feruloyltyramine (FerTyr), 4-coumaroyl-3-hydroxyagmatine, feruloylagmatine (FerAgm), N-p-coumaroylagmatine (CouAgm), terrestriamide and feruloylserotonin (FerSer), and then greatly reduced the subsequent level of damage caused by the pathogen^12^. In rice, N-p-coumaroylserotonin is induced in leaves by both *Bipolaris oryzae* infection and UV irradiation, and suppresses the growth of the bacterial pathogen *Xanthomonas oryzae* pv. *oryzicola* (*Xoo*, which causes rice leaf streak disease) with a half-inhibition concentration of 54.54 g/mL^11^. In addition, PAs also engage in herbivore defense^13^. Absence of N-caffeoylputrescine (CafPut) and dicaffeoylspermidine reduced the resistance of *Nicotiana attenuata* to herbivores. Moreover, the growth of *Manduca sexta* caterpillars was slowed after caterpillars were fed *N. attenuata* leaves treated with synthetic CafPut^14^. It has been reported that levels of N-p-coumaroylputrescine (CouPut) or FerPut in rice leaves increased following the infestation of herbivores, including by the brown planthopper (BPH) *Nilaparvata lugens* and that both of these PAs reduced the survival rate of BPH^15^. However, thus far, only a few PAs have been reported to play an important role in conferring plant resistance to herbivores.

Rice, *Oryza sativa*, one of the most important staple crops worldwide, is severely infested by herbivores^16^. Infestation of rice by insect herbivores, including white-backed planthopper (WBPH) *Sogatella furcifera* and BPH, changes levels of a variety of defense-related phytohormones, including JA, JA-Ile, SA and ethylene^17–19^. Changes in these compounds, in turn, cause rice plants to produce defense responses, such as the increased activity of trypsin protease inhibitors, the production of PAs and the release of volatiles; these responses enhance the direct and/or indirect resistance of rice to herbivores^18–20^. BPH infestation has been reported to reduce the resistance of rice to WBPH and therefore improve the performance of WBPH on rice plants^21^. Moreover, as mentioned above, BPH female adult infestation induced the production of CouPut and FerPut^15^. Yet, whether infestation of WBPH induces the production of PAs in rice, whether and which PAs are defensive against WBPH and whether the suppression of BPH infestation on the resistance of rice to WBPH is related to PA levels remain unknown.

In this study, we found that infestation by WBPH gravid females induced the production of JA, JA-Ile and H_2_O_2,_ and repressed the biosynthesis of ethylene, which subsequently enhanced levels of 11 of the tested 12 PAs; infestation by WBPH nymphs, on the other hand, did not alter levels of JA, JA-Ile, H_2_O_2_ and ethylene, and elicited accumulation of only 2 of 12 PAs. Unlike WBPH gravid female infestation, BPH gravid female infestation induced the production of only 3 PAs while suppressing the biosynthesis of 4. Bioassays revealed that 4 PAs – FerPut, FerTyr, FerAgm and N1, N10-diferuloylspermidine (DiferSpe) – were toxic to WBPH female adults. The results demonstrate that (1) the influence of herbivore infestation on the biosynthesis of PAs in rice is probably related to the profile of signaling pathways that the herbivore elicited and (2) PAs confer the resistance to WBPH on rice.

## Results

### Infestation of WBPH gravid females but not of nymphs induces the biosynthesis of JA, JA-Ile and H_2_O_2_ in rice

**Figure 1:**
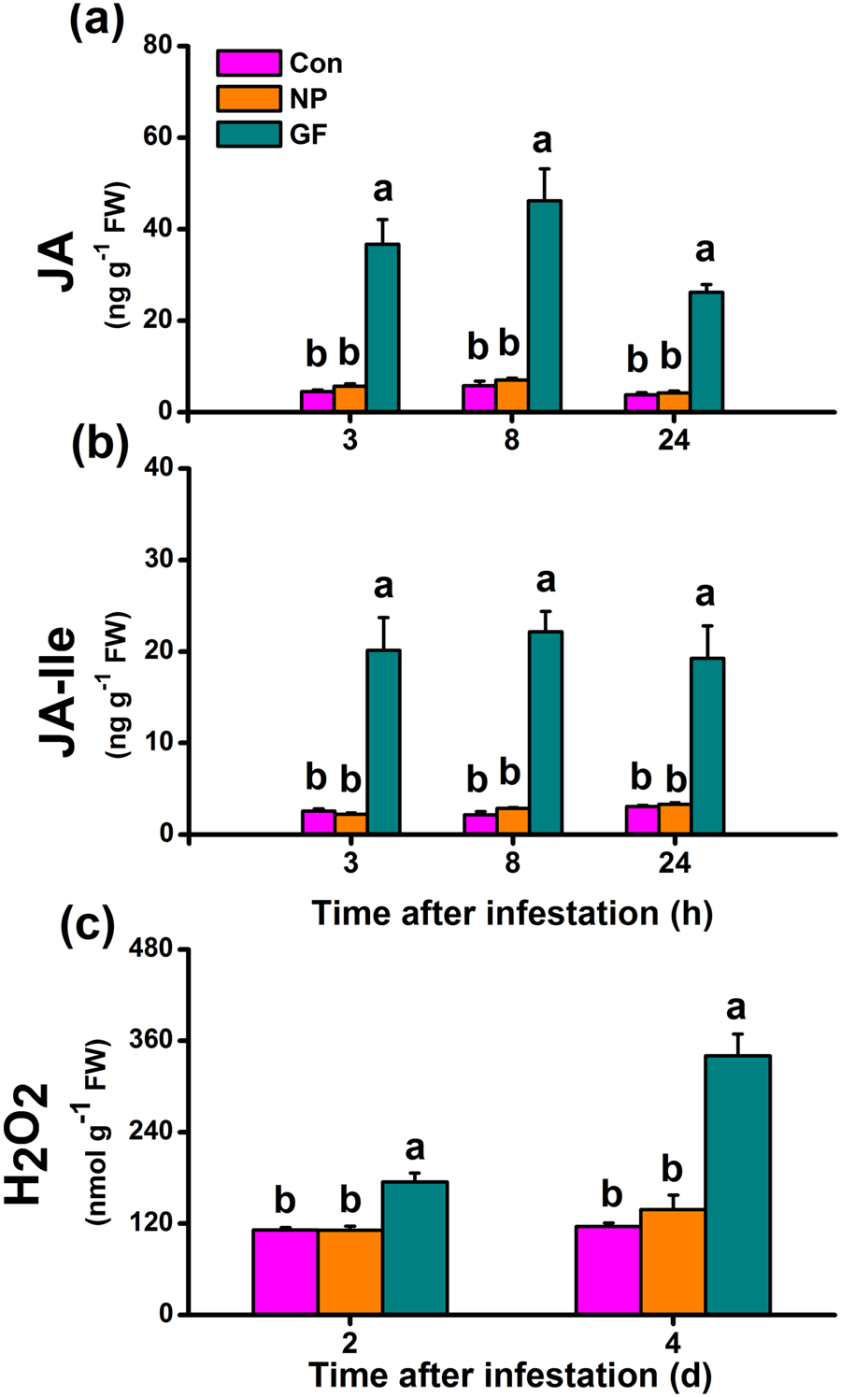
Influence of WBPH infestation on levels of JA, JA-Ile and H_2_O_2_ in rice. Mean levels (+SE, n = 5) of JA (**a**) and JA-Ile (**b**) in rice plants at 3, 8 and 24 h after exposure to WBPH infestation. Con, non-infested plants; NP, plants infested by WBPH nymphs; GF, plants infested by WBPH gravid females. Letters indicate significant differences among treatments (*P* < 0.05; Duncan’s multiple-range test).

JA, JA-Ile and H_2_O_2_ play vital roles in plant defense responses to herbivores^1–3^. Therefore, we investigated whether WBPH infestation induced the biosynthesis of these signals. We found that WBPH nymph infestation did not change levels of JA and JA-Ile in rice plants compared to in non-infested rice plants (Fig. 1). However, levels of JA and JA-Ile in rice plants at 3, 8 and 24 h after infestation by WBPH gravid females were 8.1-, 7.9-, 6.9- and 7.8-, 10.2-, 6.3-fold higher than levels of JA and JA-Ile in non-infested rice plants, respectively (Fig. 1a,b). Similarly, WBPH nymph infestation did not induce the production of H_2_O_2_ in rice at 2 and 4 d after infestation, yet WBPH gravid female infestation did significantly enhance levels of H_2_O_2_ (Fig. 1c). In summary, WBPH gravid female infestation elicited the production of JA, JA-Ile and H_2_O_2_ in rice, whereas WBPH nymph infestation had no influence on the biosynthesis of these two signals.

### Infestation of WBPH gravid females but not of nymphs suppresses the biosynthesis of ethylene in rice

**Figure 2:**
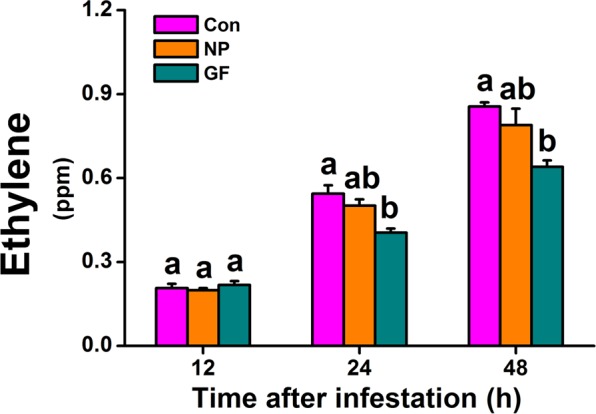
Influence of WBPH infestation on levels of ethylene in rice. Mean levels (+SE, n = 5) of ethylene released from plants at 12, 24 and 48 h after exposure to WBPH infestation. Con, non-infested plants; NP, plants infested by WBPH nymphs; GF, plants infested by WBPH gravid females. Letters indicate significant differences among treatments (*P* < 0.05; Duncan’s multiple-range test).

Ethylene-mediated signaling also plays an important role in herbivore-induced plant defenses^1,3,18^. In rice, ethylene signaling reportedly positively regulated the resistance of rice to the striped stem borer *Chilo suppressalis* but negatively mediated the resistance to BPH^18^. In this study, we observed that infestation of WBPH gravid females reduced the level of ethylene in rice: ethylene levels in rice plants infested by gravid WBPH females 24 and 48 h after infestation were 78.1% and 75.7% of those in non-infested rice plants (Fig. 2). Unlike the infestation of WBPH gravid females, the infestation of WBPH nymphs did not influence the production of ethylene in rice (Fig. 2). In summary, WBPH gravid female infestation suppressed the biosynthesis of ethylene in rice whereas WBHP nymph infestation did not.

### Infestation of WBPH gravid females and nymphs differently induces the biosynthesis of phenolamides in rice

**Figure 3:**
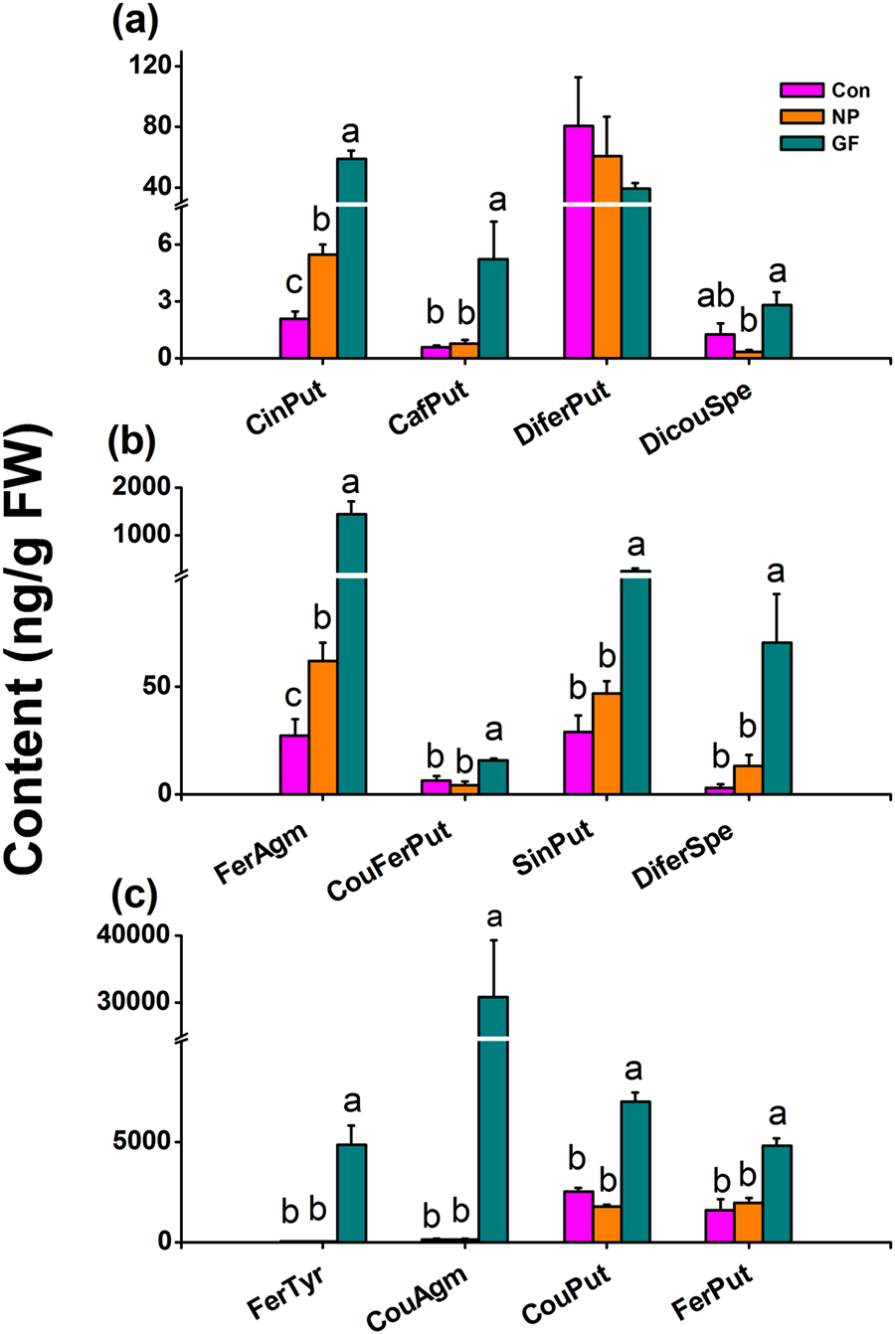
Influence of WBPH infestation on levels of phenolamides in rice. Mean levels (+SE, n = 5) of phenolamides in rice plants 4 days after they were infested by WBPH nymphs (NP), WBPH gravid females (GF) or kept non-infested (Con). CinPut, N-cinnamoylputrescine; CafPut, N-caffeoylputrescine; DiferPut, N,N′-diferuloylputrescine; DicouSpe, N1,N10-dicoumaroylspermidine; FerAgm, feruloylagmatine; CouFerPut, N-p-coumaroyl-N′-feruloylputrescine; SinPut, N-sinapoylputrescine; DiferSpe, N1,N10-diferuloylspermidine; FerTyr, N-feruloyltyramine; CouAgm, N-p-coumaroylagmatine; CouPut, N-p-coumaroylputrescine; FerPut, N-feruloylputrescine. Letters indicate significant differences among treatments (*P* < 0.05; Duncan’s multiple-range test).

WBPH gravid female infestation regulated the biosynthesis of JA, JA-Ile, H_2_O_2_ and ethylene, whereas WBPH nymph infestation did not (Figs. 1 and 2). Thus, we wanted to know if WBPH gravid female and nymph infestation also differently influences the production of PAs in rice. In PA analysis, we used 14 PA standard compounds – N-cinnamoylputrescine (CinPut), CouPut, CafPut, FerPut, CouAgm, N-sinapoylputrescine (SinPut), FerAgm, FerTyr, N-p-coumaroyl-N′-feruloylputrescine (CouFerPut), N1,N10-dicoumaroylspermidine (DicouSpe), DiferSpe, N,N′-diferuloylputrescine (DiferPut), N-sinapoylagmatine (SinAgm) and N1,N10-disinapoylspermidine (DisinSpe), 12 of which (all except SinAgm and DisinSpe) were detected in rice. When rice plants were infested by WBPH gravid females, levels of 11 of the 12 identified PAs (only DiferPut was absent) in leaf sheaths were significantly increased. CouAgm was the most induced: its level in gravid female-infested plants was 249.2-fold higher than its level in non-infested plants (Fig. 3a–c). Infestation by WBPH nymph increased levels of only two PAs, FerAgm and CinPut; levels of other PAs remained unchanged (Fig. 3a–c). Clearly, WBPH female adult and WBPH nymph infestation had different effects on the biosynthesis of PAs in rice.

### BPH infestation influences the production of PAs in rice differently than WBPH infestation

**Figure 4:**
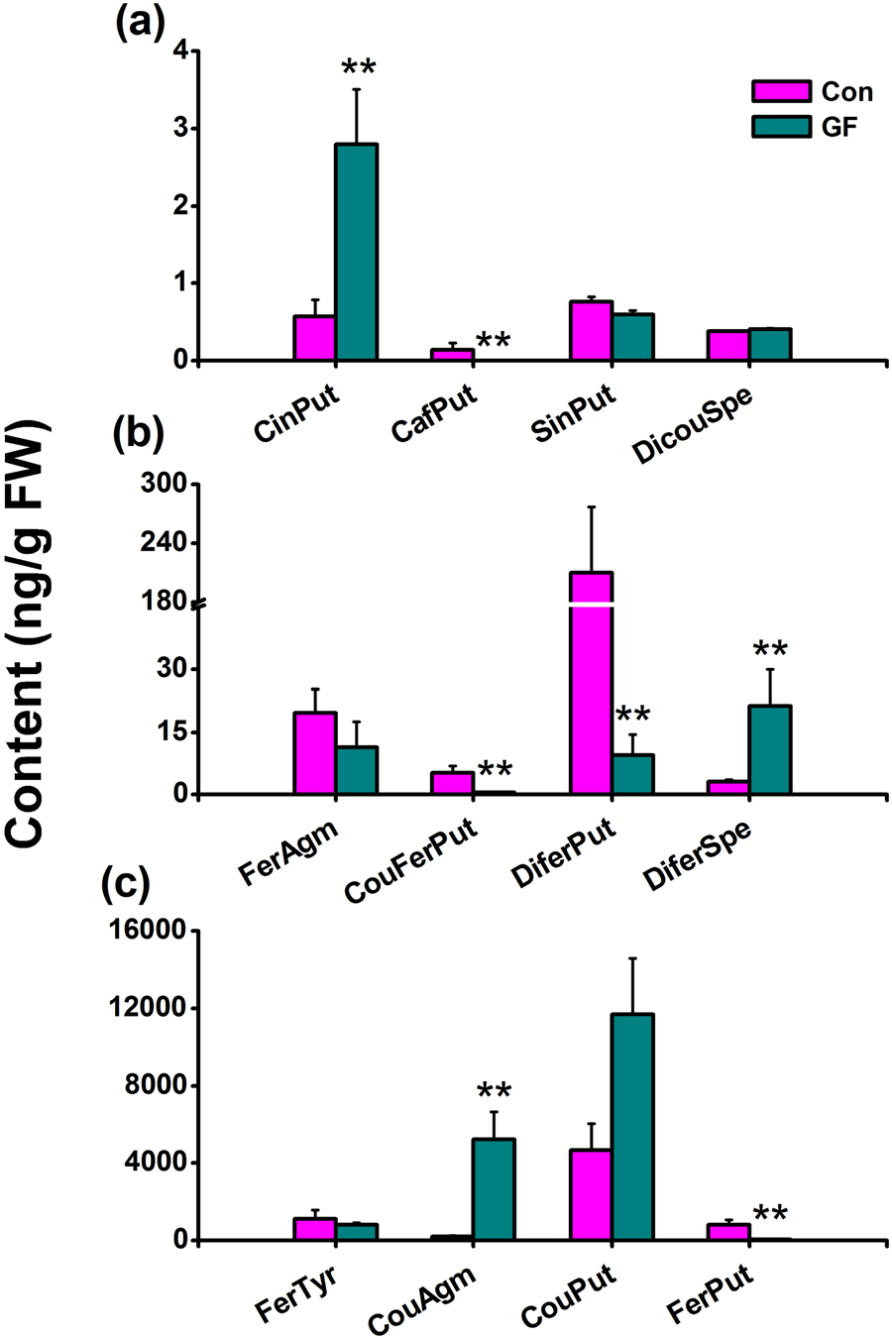
Influence of BPH infestation on phenolamides in rice. Mean levels (+SE, n = 5) of phenolamides in rice plants 3 days after they were infested by BPH gravid females (GF) or kept non-infested (Con). CinPut, N-cinnamoylputrescine; CafPut, N-caffeoylputrescine; DiferPut, N,N′-diferuloylputrescine; DicouSpe, N1,N10-dicoumaroylspermidine; FerAgm, feruloylagmatine; CouFerPut, N-p-coumaroyl-N′-feruloylputrescine; SinPut, N-sinapoylputrescine; DiferSpe, N1,N10-diferuloylspermidine; FerTyr, N-feruloyltyramine; CouAgm, N-p-coumaroylagmatine; CouPut, N-p-coumaroylputrescine; FerPut, N-feruloylputrescine. Asterisks indicate significant differences between treatments and controls (*P < 0.05; **P < 0.01; Student’s *t*-test).

Levels of CouPut and FerPut in rice leaves were reported to increase when leaves were infested by BPH female adults^15^. Here we investigated the accumulation of PAs in rice leaf sheaths, the normal feeding and oviposition site of BPH on rice plants, after these sheaths had been infested with BPH gravid females for 3 days. The results showed that BPH gravid female infestation increased levels of 3 PAs – CinPut, DiferSpe and CouAgm–in rice leaf sheaths: levels of CinPut, DiferSpe and CouAgm in leaf sheaths of plants infested by BPH gravid females were 4.9, 7.2 and 24.3-fold, respectively, higher than those in leaf sheaths of non-infested plants (Fig. 4). Moreover, compared to levels in leaf sheaths of non-infested plants, levels of 4 PAs – CafPut, CouFerPut, DiferPut and FerPut – in leaf sheaths of plants infested by gravid BPH females decreased (Fig. 4). Levels of the 5 remaining PAs did not differ between BPH gravid female-infested plants and non-infested plants. BPH gravid female infestation clearly influences the production of PAs in rice differently from WBPH gravid female infestation.

### Several PAs had a direct negative effect on the survival of WBPH female adults

**Figure 5:**
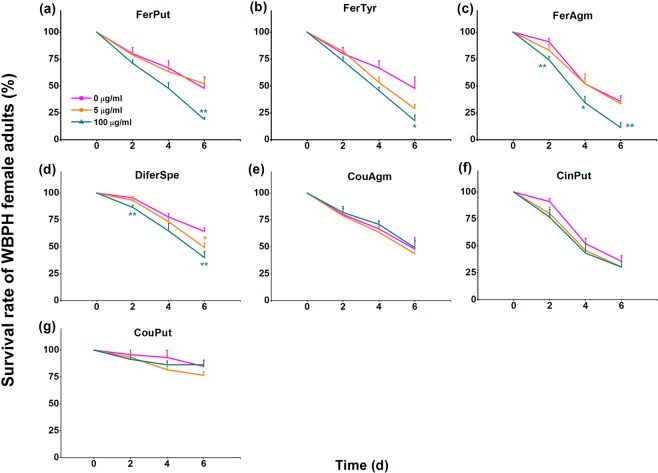
Effect of phenolamides on the survival rate of WBPH female adults. Mean survival rates (+SE, n = 7) of 15 newly emerged WBPH female adults fed on artificial diet complemented with FerPut (**a**), FerTyr (**b**), FerAgm (**c**), DiferSpe  (**d**), CouAgm (**e**), CinPut (**f**) and DiferSpe (**g**) at concentrations of 0, 5 or 100 μg/ml. FerPut, N-feruloylputrescine; FerTyr, N-feruloyltyramine; FerAgm, feruloylagmatine; CouAgm, N-p-coumaroylagmatine; CinPut, N-cinnamoylputrescine; DiferSpe, N1,N10-diferuloylspermidine; CouPut, N-p-coumaroylputrescine. Asterisks indicate significant differences between treatments and controls (*P < 0.05; **P < 0.01; Student’s *t*-test).

As two PAs have been reported to reduce the survival of BPH female adults^15^, we investigated if some of the induced PAs also negatively influenced the survival of WBPH. Seven PAs – FerPut, FerTyr, FerAgm, CouAgm, CinPut, DiferSpe and CouPut – all of which were significantly induced by WBPH gravid female infestation, were chosen to be added to artificial diet to examine their direct influence on the performance of newly emerged WBPH female adults. Six d after the start of feeding, the survival rate of WBPH female adults fed on artificial diet with FerPut and FerTyr at concentrations of 100 μg/ml significantly decreased (by 60.5% and 62.8%, respectively), compared to the survival rate of WBPH female adults fed on artificial diet without PAs (Fig. 5a,b); the survival rate of WBPH female adults fed on artificial diet with FerAgm at a concentration of 100 μg/ml significantly decreased starting at 2 d after feeding, with a maximum decrease of 68.4% at 6 d (Fig. 5c). For DiferSpe, a concentration of 5 μg/ml caused significant reductions in the survival of WBPH female adults 6 d after feeding; when the concentration of DiferSpe rose to 100 μg/ml, the survival of WBPH decreased starting 2 d after feeding, reaching a minimum at 6 d (Fig. 5d). Three PAs – CinPut, CouAgm and CouPut – had no effect on the survival rate of WBPH female adults (Fig. 5e,f). In summary, WBPH gravid female-induced FerPut, FerTyr, FerAgm and DiferSpe were toxic to WBPH.

## Discussion

In this study, we identified 12 PAs in rice and found that 11 of these PAs were induced by WBPH gravid female infestation, whereas, surprisingly, only 2 were induced by WBPH nymph infestation (Fig. 3). Bioassays revealed that 4 of the tested 7 PAs – FerPut, FerTyr, FerAgm and DiferSpe–reduced the survival of newly emerged WBPH female adults. Phytohormone analysis showed that WBPH gravid female infestation significantly increased levels of JA, JA-Ile and H_2_O_2,_ and reduced the level of ethylene in rice, whereas WBPH nymph infestation had no effect (Figs. 2, 3). These findings demonstrate that PAs play an important role in the resistance of rice to WBPH and that the infestation of rice by WBPH gravid females and nymphs induces levels of PAs, and these differ according to the defense-related signaling pathways that are elicited at the same time.

PAs have been reported to be induced by pathogen infection^22,23^, herbivore infestation^15^ and UV radiation^11^. Moreover, JA- and ethylene-mediated signaling pathways (though not the SA-mediated pathway) positively regulate the production of PAs^6,13^. We found that infestation by WBPH gravid females had a stronger influence on the biosynthesis of PAs than infestation by WBPH nymphs; this difference is probably due to different influences of WBPH gravid female infestation and WBPH nymph infestation on the production of defense-related signals, JA, JA-Ile, H_2_O_2_ and ethylene. The reason why WBPH nymph infestation and WBPH gravid female infestation have different effects on levels of JA, JA-Ile, ethylene and H_2_O_2_ is probably related to their different damage modes: nymphs just pierce and suck phloem sap, causing very little damage, whereas gravid females not only pierce and suck phloem sap but also lay eggs into leaf sheaths; oviposition (laying eggs into leaf sheaths) will result in more mechanical wounding compared to that caused by WBPH feeding^16^. Moreover, different effectors and/or elicitors derived from WBPH salivary glands/oral regurgitant and/or egg fluid may also contribute to this difference. It has been well documented that elicitors and/or effectors exist in both herbivore salivary glands/oral regurgitant and egg fluid^2,24–26^. In BPH, several elicitors and effectors from salivary glands have also been identified^27–31^. Interestingly, we also observed that WBPH and BPH gravid females induced PAs differently (Fig. 4): BPH infestation reduced levels of 4 PAs in rice sheaths, whereas these were induced by WBPH infestation, and one of these 4 PAs, FerPut, was toxic to WBPH; in addition, levels of 3 BPH-induced PAs – CinPut, DiferSpe and CouAgm – were generally lower than those induced by WBPH (Fig. 3). Although both WBPH gravid female infestation and BPH gravid female infestation have been reported to induce the production of JA, JA-Ile and H_2_O_2_, and to suppress the production of ethylene, the profile of these signals, including the level and timing of their emission, differs between the two insect species^32^. Hence, the herbivore-induced biosynthesis of PAs in rice seems to be shaped primarily by the specific profile of defense-related signals elicited by the herbivore.

PAs have been reported to enhance the resistance of plants to pathogens and herbivores via both reinforced cell walls, which reduce the digestibility of herbivores and invasion of pathogens, and direct toxicity for pathogens and herbivores^9,15,22,33^. We found that the survival rate of WBPH female adults fed on artificial diets complemented with certain concentrations of each of the following 4 PAs – FerPut (100 μg/mL), FerTyr (100 μg/mL), FerAgm (100 μg/mL) and DiferSpe (5 and 100 μg/mL) – decreased significantly. This suggests that some PAs are toxic to WBPH, just as they are to BPH adults^15^. However, the effect of PAs on different herbivores is not identical: CouPut, for example, has been found to reduce the survival rate of BPH adults^15^ but had no negative influence on WBPH adults (Fig. 5). Although the levels of these toxic PAs determined here were lower than the levels used in bioassays, it should be emphasized that both BPH and WBPH feed phloem sap where levels of these PAs may be higher than in other tissues, a direction worthy for future analysis. It may be that levels of these PAs in plant phloem may be higher than in other tissues, a direction for future analysis. Moreover, Alamgir *et al*.^15^ found that the level of FerPut in rice leaves under heavy BPH infestation could be up to 60 μg/g FM; this level is comparable to the level of FerPut that were effective we found in bioassays. Taken together, these findings demonstrate that PAs enhance the resistance of rice to WBPH.

Previous investigations have found that BPH infestation can improve the performance of WBPH, including enhancing WBPH survival rates and feeding amounts^21^. Considering that PAs are toxic to WBPH, the results observed in this study–namely, that fewer PAs were induced by BPH infestation than by WBPH infestation, and lower levels of PAs in were found in BPH-infested plants than in WBPH-infested plants – may be part of the explanation for the difference. Interestingly, we found that the effect of BPH infestation on the biosynthesis of PAs was different from the result reported in Alamgir *et al*.^15^. They showed that both CouPut and FerPut can be induced by BPH infestation, whereas we observed that BPH infestation reduced the level of FerPut and did not or only marginally induce the production of CouPut (Fig. 4). This discrepancy may be related to the different rice varieties used and rice tissues sampled: in the study of Alamgir *et al*.^15^, leaves from the japonica variety Nipponbare were sampled; we sampled leaf sheaths from the japonica variety XS110. Further research is needed to elucidate this issue.

In summary, rice PAs respond differently to WBPH gravid female infestation, WBPH nymph infestation and BPH gravid female infestation, probably because these herbivore infestations induced different profiles of defense-related signals. Like the negative effects of PAs on survival rates of BPH reported in Alamgir *et al*.^15^, we also found that PAs reduced survival rates of WBPH female adults. Given that PAs in rice can be induced by pathogens, such as blast fungus *Magnaporthe oryzae* and rice brown spot fungus *B. oryzae*^34^, and other insect pests, such as the lawn armyworm *Spodoptera mauritia* and the rice skipper *Parnara guttata* larvae^15^, and given that PAs are toxic to *B. oryzae*, *Xoo*^34^, WBPH and BPH, we expect them to play a vital role in interactions among rice, insect pests and pathogens. Elucidating the functions of PAs by using genetically modified techniques may decipher some of these complicated interactions.

## Methods

### Plant growth and insect rearing

The rice used in this study was a *japonica* type variety XS110. Plants were grown used the same method as described in Huangfu *et al*.^35^. Briefly, pre-germinated seeds of XS110 were sown in plastic bottles (diameter 8 cm, height 10 cm) in a greenhouse at 28 ± 2 °C and 14 h photophase, and after 10 days, the seedlings were transferred to 20-L hydroponic boxes with a rice nutrient solution^36^. Twenty-five days later, seedlings were transplanted to individual 500 mL hydroponic opaque plastic pots (diameter 8 cm, height 10 cm), each with one plant. Plants were used for experiments 4–5 days after transplanting.

WBPH and BPH colonies were originally obtained from rice fields in Hangzhou, China, and maintained on rice seedlings of TN1, a variety susceptible to WBPH and BPH, in a controlled climate room that was maintained at 26 ± 2 °C, 12 h photophase and 80% relative humidity.

### Plant treatments

For WBPH treatment, plants (one per pot) were individually infested with 20 WBPH third-instar nymphs (NP) or 15 WBPH gravid females (GF); plants and insects were confined in glass cages (diameter, 4 cm; height, 8 cm; with 48 small holes). For BPH treatment, plants (one per pot) were individually infested in the glass cages with 15 BPH gravid females (GF). Plants with empty glass cages were used as controls (Con).

### JA, JA-Ile, SA and ET analysis

Rice plants (one per pot) were randomly assigned to the following treatments: WBPH nymph, WBPH gravid female and control. For JA, JA-Ile, and SA analysis, leaf sheaths of plants were harvested at 3, 8 and 24 h after the start of treatment. Samples were ground with liquid nitrogen, and JA, JA-Ile and SA were extracted with ethyl acetate spiked with labeled internal standards ([^2^D_6_]JA, [^2^D_6_]JA-Ile, and [^2^D_4_]SA), and then analyzed with HPLC/mass spectrometry/mass spectrometry following the method described by Lu *et al*.^18^. Each plant treatment at each time interval was replicated five times. For ET analysis, plants infested with WBPH nymphs or gravid females and control plants were covered with sealed glass cylinders (diameter, 4 cm; height, 50 cm). ET production was determined at 12, 24 and 48 h after treatment using the same method as described by Lu *et al*.^18^. Each plant treatment at each time interval was replicated ten times.

### PA content determination

The experiments were performed twice using two batches of plants. For the first batch of plants, plants were randomly assigned to treatments as follows: WBPH nymph, WBPH gravid female and control. For the second batch of plants, plants were randomly assigned to BPH gravid female and control treatment. Rice sheaths were harvested 3 d after BPH or 4 d after WBPH infestation, and each treatment was replicated 5 times.

Samples (100 mg each) were ground in liquid nitrogen, and PAs were extracted and analyzed using the method as described in Alamgir *et al*.^15^ with minor modifications. Briefly, homogenized samples were individually mixed with 1 ml of 80% ethanol (1 ml/100 mg fresh weight tissue). All samples were then vortexed for 5 min, extracted by ultrasonic for 20 min and centrifuged at 13,000 rpm for 20 min at 4 °C. The supernatants were collected and measured using HPLC-MS-MS (Agilent Technologies, Santa Clara, CA, USA) equipped with a Zorbax SB-C_18_ column [2.1 mm I·D × 150 mm, (3.5 µm), Agilent Technologies] at 35 °C. The injected volume of sample was 10 μl. The gradient mobile phase consisted of 0.25% formic acid in water (solvent A) and acetonitrile (solvent B) at a constant flow rate of 0.3 ml min^−1^. A linear gradient profile with the following proportions (v/v) of solvent B was applied: 0 to 7 min, 5% of B; 7 to 9 min, 5% to 55% of B; 9–11 min, 55% to 95% of B. Multiple reaction monitoring (MRM) mode was used to quantify PAs. MRM conditions and compound quantification were optimized using the 14 standard PAs. The m/z of CinPut was 219.1/131.0; the m/z of CouPut was 235.1/147.0; the m/z of CafPut was 251.3/163.0; the m/z of FerPut was 265.3/177.1; the m/z of CouAgm was 277.11/147.0; the m/z of SinPut was 295.1/207.1; the m/z of FerAgm was 307.21/177.1;the m/z of FerTyr was 314.4/177.1; the m/z of SinAgm was 337.2/207.1; the m/z of CouFerPut was 411.2/177.0; the m/z of DicouSpe was 438.0/147.0; the m/z of DiferPut was 441.2/177.0; the m/z of DiferSpe was 498.2/234.1; the m/z of DisinSpe was 558.3/264.1. All standard PAs used in the experiments are chromatographic grade and obtained from Hangzhou Chemipanda Bio-Tech Co., Ltd (China, Hangzhou).

### Direct effects of PAs on WBPH

Each of the 7 PAs – FerPut, FerTyr, FerAgm, CouAgm, CinPut, DiferSpe and CouPut – was dissolved in methanol (1 mg per ml methanol) and then added to artificial diet at a final concentration of 5 or 100 µg/ml^37^. Fifteen newly emerged WBPH female adults were fed on artificial diet containing one of the 8 PAs at a concentration of 5 or 100 mg L^−1^ in a 30 ml double-ended open glass cylinder (diameter 2 cm, length 9 cm) as described in Fu *et al*.^38^. Controls were fed on artificial diet without PAs. Each treatment was replicated seven times. The number of adults alive was recorded every day.

### Statistical analysis

Differences in data in different WBPH and BPH treatments were analyzed by Duncan’s multiple range test (or Student’s *t*-test for comparing two treatments). All tests were carried out with Statistica 6 (Statistica, SAS Institute Inc., Cary, NC, USA).

## References

[1] Erb M, Meldau S, Howe GA (2012). Role of phytohormones in insect-specific plant reactions. Trends Plant Sci..

[2] Hilker M, Fatouros NE (2015). Plant responses to insect egg deposition. Annu. Rev. Entomol..

[3] Schuman MC, Baldwin IT (2016). The layers of plant responses to insect herbivores. Annu. Rev. Entomol..

[4] Engelberth, J. Plant resistance to insect herbivory in *Biocommunication of Plants* (eds Witzany & Baluska) 303–326 (Springer (2012).

[5] Bassard JE, Ullmann P, Bernier F, Werck-Reichhart D (2010). Phenolamides: bridging polyamines to the phenolic metabolism. Phytochemistry.

[6] Kusano T, Yamaguchi K, Berberich T, Takahashi Y (2007). Advances in polyamine research in 2007. J. Plant Res..

[7] Moschou PN, Paschalidis KA, Roubelakis-Angelakis KA (2008). Plant polyamine catabolism. Plant Signal. Behav..

[8] Campos L, Lisón P, López-Gresa MP, Rodrigo I, Bellés JM (2014). Transgenic tomato plants overexpressing tyramine N-hydroxycinnamoyltransferase exhibit elevated hydroxycinnamic acid amide levels and enhanced resistance to *Pseudomonas syringae*. Mol. Plant Microbe. Interact..

[9] Macoy DM, Kim W, Lee SY, Kim MG (2015). Biotic stress related functions of hydroxycinnamic acid amide in plants. J. Plant Biol..

[10] Macoy DM, Kim W, Lee SY, Kim MG (2015). Biosynthesis, physiology, and functions of hydroxycinnamic acid amides in plants. Plant Biotechnol. Rep..

[11] Park HL (2014). Antimicrobial activity of UV-induced phenylamides from rice leaves. Molecules.

[12] Yogendra KN, Pushpa D, Mosa KA, Kushalappa AC, Mosquera T (2014). Quantitative resistance in potato leaves to late blight associated with induced hydroxycinnamic acid amides. Funct. Integr. Genomic..

[13] Gaquerel E, Gulati J, Baldwin I (2014). Revealing insect herbivory-induced phenolamide metabolism: from single genes to metabolic network plasticity analysis. Plant J..

[14] Kaur H, Heinzel N, Schöttner M, Baldwin I, Galis I (2010). R2R3-NaMYB8 regulates the accumulation of phenylpropanoid-polyamine conjugates, which are essential for local and systemic defense against insect herbivores in *Nicotiana attenuata*. Plant Physiol..

[15] Alamgir KM (2016). Systematic analysis of rice (*Oryza sativa*) metabolic responses to herbivory. Plant Cell Environ..

[16] Pathak, M. D. & Khan, Z. R. *Insect Pests of Rice*. (1994).

[17] Lou, Y.; Lu, J. & Li, J. Herbivore-induced defenses in rice and their potential application in rice planthopper management in *Rice* Planthoppers (eds Heong, K.L., Cheng, J., Escalada, M.M) 91-116 (Springer Netherlands (2015).

[18] Lu J, Li J, Ju H, Liu X, Lou Y (2014). Contrasting effects of ethylene biosynthesis on induced plant resistance against a chewing and a piercing-sucking herbivore in rice. Mol. Plant..

[19] Zhou G (2009). Silencing OsHI-LOX makes rice more susceptible to chewing herbivores, but enhances resistance to a phloem feeder. Plant J..

[20] Kachroo A, Kachroo P (2007). Salicylic acid-, jasmonic acid- and ethylene-mediated regulation of plant defense signaling. Genet. Eng..

[21] Cao T, Backus EA, Lou Y, Cheng J (2013). Feeding-induced interactions between *Nilaparvata lugens* and *Laodelphax striatellus* (hemiptera: delphacidae): effects on feeding behavior and honeydew excretion. Environ. Entomol..

[22] McLusky SR (1999). Cell wall alterations and localized accumulation of feruloyl-3′-methoxytyramine in onion epidermis at sites of attempted penetration by botrytis allii are associated with actin polarisation, peroxidase activity and suppression of flavonoid biosynthesis. Plant J..

[23] Yogendra KN, Kushalappa AC, Sarmiento F, Rodriguez E, Mosquera T (2015). Metabolomics deciphers quantitative resistance mechanisms in diploid potato clones against late blight. Funct. Plant Biol..

[24] Acevedo FE, Rivera-Vega LJ, Chung SH, Ray S, Felton GW (2015). Cues from chewing insects - the intersection of DAMPs, HAMPs, MAMPs and effectors. Curr. Opin. Plant Biol..

[25] Shinya T (2016). Modulation of plant defense responses to herbivores by simultaneous recognition of different herbivore-associated elicitors in rice. Sci. Rep..

[26] Uemura T, Arimura G (2019). Current opinions about herbivore-associated molecular patterns and plant intracellular signaling. Plant Signal. Behav..

[27] Huang H (2015). A salivary sheath protein essential for the interaction of the brown planthopper with rice plants. Insect Biochem. Molec..

[28] Ji R (2017). A salivary endo-b-1,4-glucanase acts as an effector that enables the brown planthopper to feed on rice. Plant Physiol..

[29] Rao W (2019). Secretome analysis and in planta expression of salivary proteins identify candidate effectors from the brown planthopper *Nilaparvata lugens*. Mol. Plant Microbe Interact..

[30] Shangguan X (2018). A mucin-like protein of planthopper is required for feeding and induces immunity response in plants. Plant Physiol..

[31] Ye W (2017). A salivary EF-hand calcium-binding protein of the brown planthopper *Nilaparvata lugens* functions as an effector for defense responses in rice. Sci. Rep..

[32] Liu X, Lou Y (2018). Comparison of the defense responses in rice induced by brown planthopper *Sogatella furcifera* (Horváth) and white-backed planthopper *Sogatella furcifera* (Horváth). J. Plant Prot..

[33] Passardi F, Penel C, Dunand C (2004). Performing the paradoxical: how plant peroxidases modify the cell wall. Trends Plant Sci..

[34] Shabana YM, Abdel-Fattah GM, Ismail AE, Rashad YM (2008). Control of brown spot pathogen of rice (*Bipolaris oryzae*) using some phenolic antioxidants. Braz. J. Microbiol..

[35] Huangfu JY (2016). The Transcription Factor OsWRKY45 Negatively Modulates the Resistance of Rice to the Brown Planthopper Nilaparvata lugens. Int. J. Mol. Sci..

[36] Yoshida, S., Forno, D.A., Cock, J.H. & Gomez, K.A. Laboratory Manual for Physiological Studies of Rice. Manila: International Rice Research Institute (1976).

[37] Chen X (2018). Furan carbonylhydrazones-derived elicitors that Induce the resistance of rice to thebrown planthopper *Nilaparvata lugens*. Phytochem. Lett..

[38] Fu Q, Zhang Z, Hu C, Lai F, Sun Z (2001). A chemically defined diet enables continuous rearing of the brown planthopper, *Nilaparvata lugens* (Stal) (homoptera: delphacidae). Appl. Entomol. Zool..

